# Molecular mechanisms that regulate export of the planar cell-polarity protein Frizzled-6 out of the endoplasmic reticulum

**DOI:** 10.1074/jbc.RA120.012835

**Published:** 2020-05-06

**Authors:** Xiao Tang, Lina Zhang, Tianji Ma, Mo Wang, Baiying Li, Liwen Jiang, Yan Yan, Yusong Guo

**Affiliations:** 1 Division of Life Science, The Hong Kong University of Science and Technology, Hong Kong, China; 2 School of Life Sciences, Centre for Cell & Developmental Biology and State Key Laboratory of Agrobiotechnology, The Chinese University of Hong Kong, Shatin, New Territories, Hong Kong, China; 3 Hong Kong University of Science and Technology Shenzhen Research Institute, Shenzhen, China

**Keywords:** cargo sorting, coat protein complex II (COPII), planar cell polarity proteins, secretory transport pathway, Frizzled class receptor 6 (FZD6), transmembrane signaling, secretion-associated Ras-related GTPase 1A (SAR1A), cadherin EGF LAG seven-pass G-type receptor 1 (CELSR1), polybasic motif, trafficking, protein sorting, endoplasmic reticulum (ER), Golgi, vesicles

## Abstract

Planar cell polarity (PCP) is a process during which cells are polarized along the plane of the epithelium and is regulated by several transmembrane signaling proteins. After their synthesis, these PCP proteins are delivered along the secretory transport pathway to the plasma membrane, where they perform their physiological functions. However, the molecular mechanisms that regulate PCP protein transport remain largely unclear. Here, we found that the delivery of a PCP protein, Frizzled-6, to the cell surface is regulated by two conserved polybasic motifs: one located in its first intracellular loop and the other in its C-terminal cytosolic domain. We observed that the polybasic motif of Frizzled is also important for its surface localization in the *Drosophila* wing. Results from a mechanistic analysis indicated that Frizzled-6 packaging into vesicles at the endoplasmic reticulum (ER) is regulated by a direct interaction between the polybasic motif and the Glu-62 and Glu-63 residues on the secretion-associated Ras-related GTPase 1A (SAR1A) subunit of coat protein complex II (COPII). Moreover, we found that newly synthesized Frizzled-6 is associated with another PCP protein, cadherin EGF LAG seven-pass G-type receptor 1 (CELSR1), in the secretory transport pathway, and that this association regulates their surface delivery. Our results reveal insights into the molecular machinery that regulates the ER export of Frizzled-6. They also suggest that the association of CELSR1 with Frizzled-6 is important, enabling efficient Frizzled-6 delivery to the cell surface, providing a quality control mechanism that ensures the appropriate stoichiometry of these two PCP proteins at cell boundaries.

## Introduction

Planar cell polarity (PCP) is defined as a process in which cells are polarized along the plane of the epithelium. PCP plays essential roles in polarized patterning of epithelial structures, and it is also essential for a variety of coordinated cell behaviors, such as the coordinated beating of cilia in the trachea and convergent extension in developing vertebrate embryos (1).

PCP is regulated by a group of evolutionarily conserved core transmembrane proteins, including Frizzled, Van Gogh (Vang), and members in the family of cadherin EGF LAG seven-pass G-type receptors (Celsr) (2). After their synthesis on ribosomes, these PCP proteins are delivered along the secretory transport pathway to the plasma membrane, where they adopt characteristic asymmetric localizations on opposing cellular boundaries to perform their cellular functions (1). How newly synthesized transmembrane PCP proteins are delivered to the plasma membrane and whether the surface delivery process contributes to their asymmetric localizations on cell boundaries remain largely unclear. Moreover, many PCP proteins interact with each other. Evidence indicates that the intercellular interactions among PCP proteins at cell-cell junctions can stabilize their specific asymmetric localizations and regulate the endocytosis process (1–6). However, it remains unclear as to how these interactions also regulate the surface delivery of transmembrane PCP proteins along the secretory transport pathway.

We have previously demonstrated that the export of two PCP proteins, Vangl2 and Frizzled-6 (Fzd6), out of the *trans*-Golgi network (TGN) are mediated by different sorting mechanisms. TGN export of Vangl2 is mediated by an Arf family protein, Arfrp1, and the clathrin-associated adaptor complex-1 (AP-1). Arfrp1 mediates the recruitment of AP-1 to the TGN membranes and promotes AP-1 to interact with the tyrosine sorting motifs on Vangl2, thereby packaging Vang2 into transport vesicles (7). Disrupting the function of AP-1 affects the polarized pattern of PCP proteins and induces defects in PCP signaling processes in *Drosophila* wing (8). TGN export of Fzd6 depends on another clathrin adaptor, epsinR (9). EpsinR forms a stable complex with clathrin, and this complex interacts with the polybasic sorting motif on the C-terminal cytosolic domain of Fzd6 to mediate the packaging of Fzd6 into transport vesicles (9). Vangl2 and Fzd6 have been shown to be packaged into separate vesicles, presumably because of differential sorting mechanisms (9). Superresolution imaging analysis has demonstrated that Vangl2 and Fzd6 are spatially segregated and associated with AP-1 and epsinR, respectively, when exiting the TGN (10). We propose that polarized post-Golgi trafficking of Fzd6- or Vangl2-enriched vesicles contributes to their asymmetric localization.

The ER is an important station in the secretory transport pathway. ER export of Vangl2 is regulated by the COPII subunit Sec24B, which stimulates the packaging of Vangl2 into COPII vesicles (11). Disrupting the function of Sec24B causes abnormal subcellular localizations of Vangl2 in the spinal cord of mouse embryos and induces defects in neural tube closure and the orientation of cochlear hair cells (11). An ER-localized protein, Shisa, interacts with the immature glycosylated form of Fzd within the ER in *Xenopus* embryos (12). This interaction causes ER retention of Frizzled proteins, thereby inhibiting Frizzled-mediated canonical Wnt signaling events (12). It remains unclear whether a similar ER retention mechanism functions to regulate the noncanonical Wnt/PCP signaling and how Frizzled receptors are recognized by the COPII machinery to be exported out of the ER.

Here, we have analyzed the molecular mechanisms regulating ER export of Fzd6. We identified several motifs in Fzd6 that are important for exporting Fzd6 out of the ER. A polybasic motif located on its first intracellular loop directly interacts with the E62, E63 residues on the COPII subunit, Sar1A, and regulates the packaging of Fzd6 into COPII vesicles. In addition, Fzd6 and a member of the Celsr family, Celsr1, are associated with each other in the early secretory transport pathway, and this association promotes the surface delivery of Fzd6. Our study gives insight into the molecular machinery that regulates ER export of Fzd6 and demonstrates that the association of Celsr1 with Fzd6 regulates the anterograde trafficking of Fzd6 along the secretory transport pathway.

## Results

### The polybasic motif in Fzd6 is important for the packaging of Fzd6 into COPII vesicles

**Figure 1.**
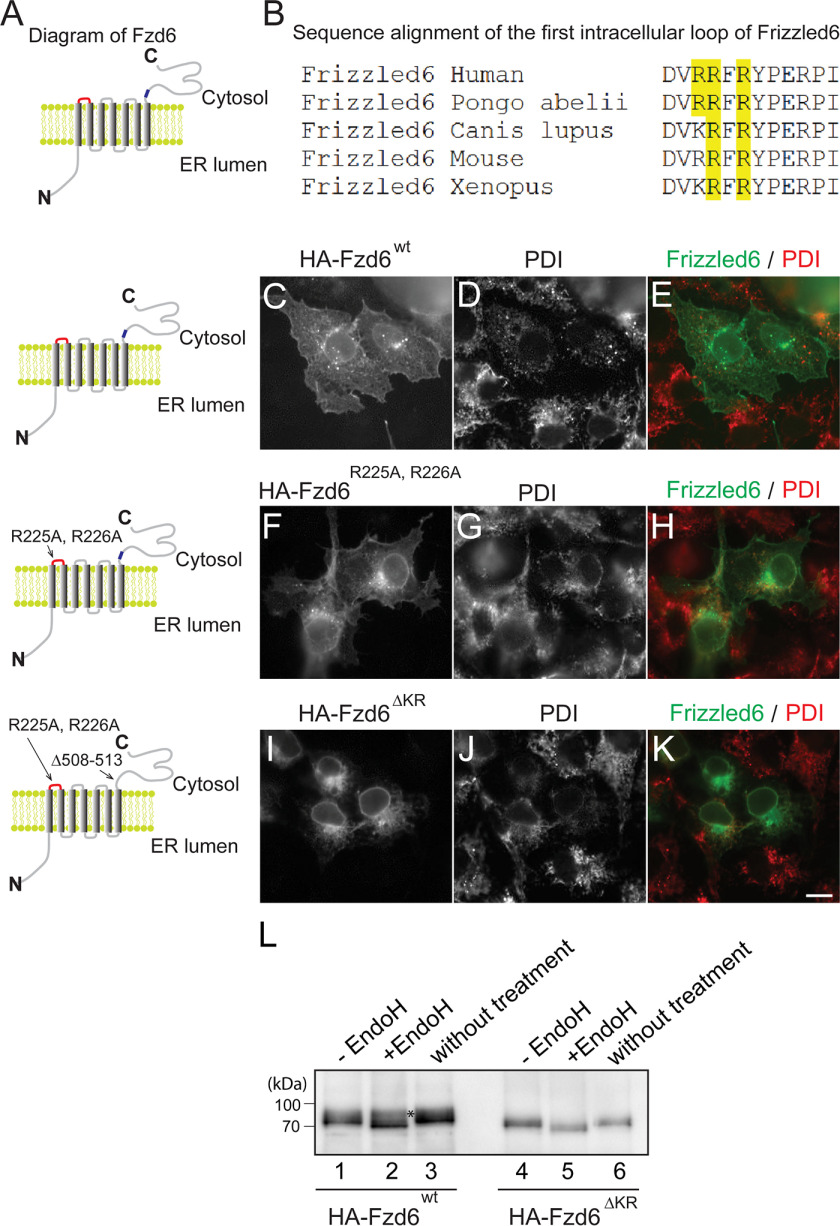
**ER export of Fzd6 depends on its polybasic motifs.** *A*, diagrams of wild-type Fzd6 and Fzd6 bearing mutations in the polybasic motifs. *Red* indicates the [R/K]RFR motif in the first intracellular loop, and *blue* indicates the C-terminal polybasic motif. *B*, sequence alignment of the first intracellular loop of Fzd6 from different species indicates that the Fzd6 contains a conserved polybasic motif in this region. *C–K*, COS7 cells were transfected with WT HA-Fzd6 (*C–E*) and HA-Fzd6 bearing mutations in its polybasic motifs (HA-Fzd6^R225A, R226A^ or HA-Fzd6^ΔKR^) (*F–K*). At day 1 after transfection, the cells were analyzed by immunofluorescence. *Size bar*, 10 μm. *L*, HA-Fzd6 or HA-Fzd6^ΔKR^ immune precipitated from COS7 cell lysates was untreated (*lanes 3 and 6*) or incubated with a reaction mixture in the presence or absence of Endo-H (*lanes 1–2 and 4–5*) and then analyzed by immunoblotting.

We previously reported that a highly conserved polybasic motif, KRNRKR, in the juxtamembrane region of the Fzd6 C-terminal cytosolic domain is important for its TGN export process (Fig. 1*A*, highlighted in *blue*) (9). In addition to this motif, there is another conserved polybasic motif, [R/K]RFR, in the first intracellular loop of Fzd6 (Fig. 1*A*, highlighted in *red*, Fig. 1*B*). Wild-type HA-tagged Fzd6 (HA-Fzd6^WT^) was partially localized at the plasma membrane (Fig. 1*C*–*E*). A mutant version of HA-Fzd6, in which the first and second arginine residues in the first intracellular loop of Fzd6 were mutated to alanine (HA-Fzd6^R225A, R226A^), was also partially localized at the plasma membrane (Fig. 1*F*–*H*). We generated another mutant version of Fzd6, in which the first and second arginine residues in the first intracellular loop of Fzd6 were mutated to alanine and the C-terminal polybasic motif was depleted (HA-Fzd6^Δ508-513, R225A, R226A^, or HA-Fzd6^ΔKR^). We found that HA-Fzd6^ΔKR^ accumulated in the ER with no detectable surface pattern (Fig. 1*I*–*K*). WT HA-Fzd6 immunoprecipitated from COS7 cell lysates was partially resistant to endoglycosidase H (Endo H) digestion (Fig. 1*L*, *lane 2, asterisk*). In contrast, HA-Fzd6^ΔKR^ immune precipitated from COS7 cell lysates was sensitive to Endo H digestion (Fig. 1*L*, compare *lanes 4 and 5*), suggesting that HA-Fzd6^ΔKR^ was not processed by the *medial*- and *trans*-Golgi resident glycosylation enzymes. These results indicate that ER export of Fzd6 is redundantly regulated by two polybasic motifs: one located in its first intracellular loop and the other located in its C-terminal cytosolic domain.

**Figure 2.**
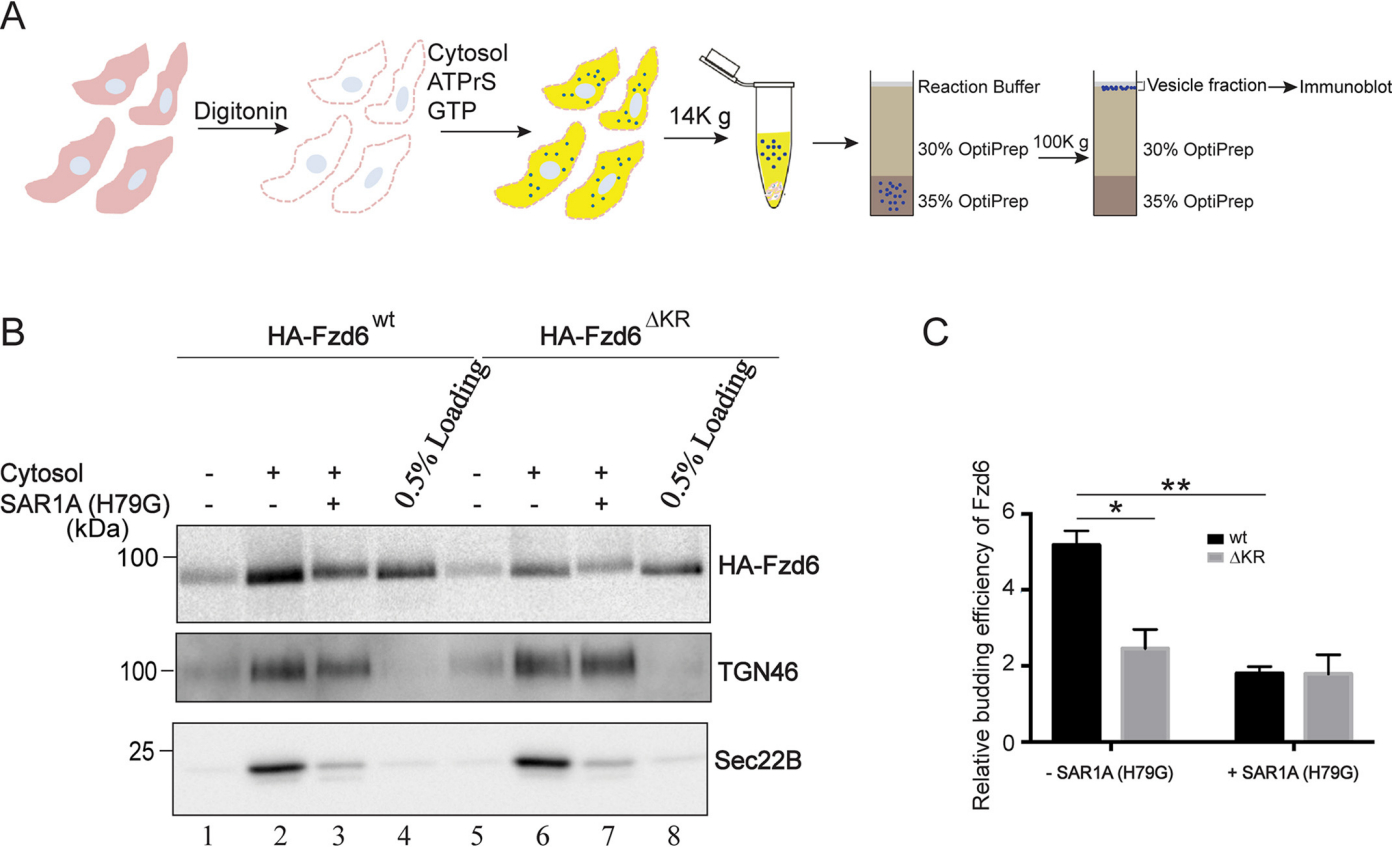
**The polybasic motif in Fzd6 is important for the packaging of Fzd6 into COPII vesicles.** *A*, a diagram showing the *in vitro* assay that reconstitutes vesicle release from HEK293T cells. *B–C*, the vesicle formation assay was performed using HEK293T cells expressing WT HA-Fzd6 (HA-Fzd6^WT^) or HA-Fzd6 deleted of the polybasic motifs (HA-Fzd6^ΔKR^). Vesicle fractions were analyzed by immunoblotting with the antibodies as indicated (*B*). The budding efficiency of HA-Fzd6^WT^ and HA-Fzd6^△KR^ from the vesicle formation assay performed with and without purified Sar1A (H79G) was quantified (*n* = 3, mean ± S.D.) (*C*). The quantification is normalized to the level of Fzd6 in the experimental group performed without cytosol. *, *p* < 0.05; **, *p* < 0.01.

To analyze whether ER accumulations of Fzd6^ΔKR^ were caused by defective ER export or by enhanced ER retrieval, we reconstituted *in vitro* vesicular release of Fzd6 in HEK293T cells. The vesicle formation assay that reconstitutes ER export of cargo proteins has been well established (11, 13, 14). In this reconstitution assay, HEK293T cells overexpressing Fzd6^WT^ or Fzd6^ΔKR^ were treated with digitonin to permeabilize the plasma membrane (Fig. 2*A*). The semi-intact cells then were incubated with an ATP regeneration system (ATPrS), GTP, and rat liver cytosol at 32 °C for 1 h. Subsequently, the cell debris and large membranes were removed by medium-speed centrifugation. The released vesicles were isolated by flotation, and the vesicles in the top fraction were analyzed by immunoblotting (Fig. 2*A*). The following two cargo proteins were used to monitor ER export and TGN export, respectively: Sec22B, which is a tSNARE cycling between the ER and the Golgi (15), and TGN46, which is a TGN-localized protein cycling between the TGN and the plasma membrane (16).

We found that Fzd6^WT^, Sec22B, and TGN46 were efficiently packaged into transport vesicles in the presence of cytosol (Fig. 2*B*, compare *lanes 1 and 2*). The efficiency of the packaging of Fzd6 into transport vesicles was significantly reduced when the vesicle formation assay was performed in the presence of a GTP hydrolysis-defective mutant form of Sar1A, Sar1A (H79G) (Fig. 2*B*, compare *lanes 2 and 3*, Fig. 2*C*). In contrast, Sar1A (H79G) did not interfere with vesicular release of TGN46 (Fig. 2*B*). This analysis indicates that a major fraction of Fzd6 detected in the vesicle fraction was in COPII vesicles. The efficiency of vesicular release of Fzd6^ΔKR^ was significantly lower than that of Fzd6^WT^ (Fig. 2*B*, compare *lanes 2 and 6*, Fig. 2*C*). In addition, Sar1A (H79G) did not show an obvious inhibition on the efficiency of vesicular release of Fzd6^ΔKR^ (Fig. 2*B*, compare *lanes 6 and 7*, Fig. 2*C*). These results are consistent with the immunofluorescence analysis, which indicates that mutating the polybasic motifs causes defects of the packaging of Fzd6 into COPII vesicles.

### The polybasic motif in Fzd6 directly interacts with the E62 and E63 residues on Sar1A

**Figure 3.**
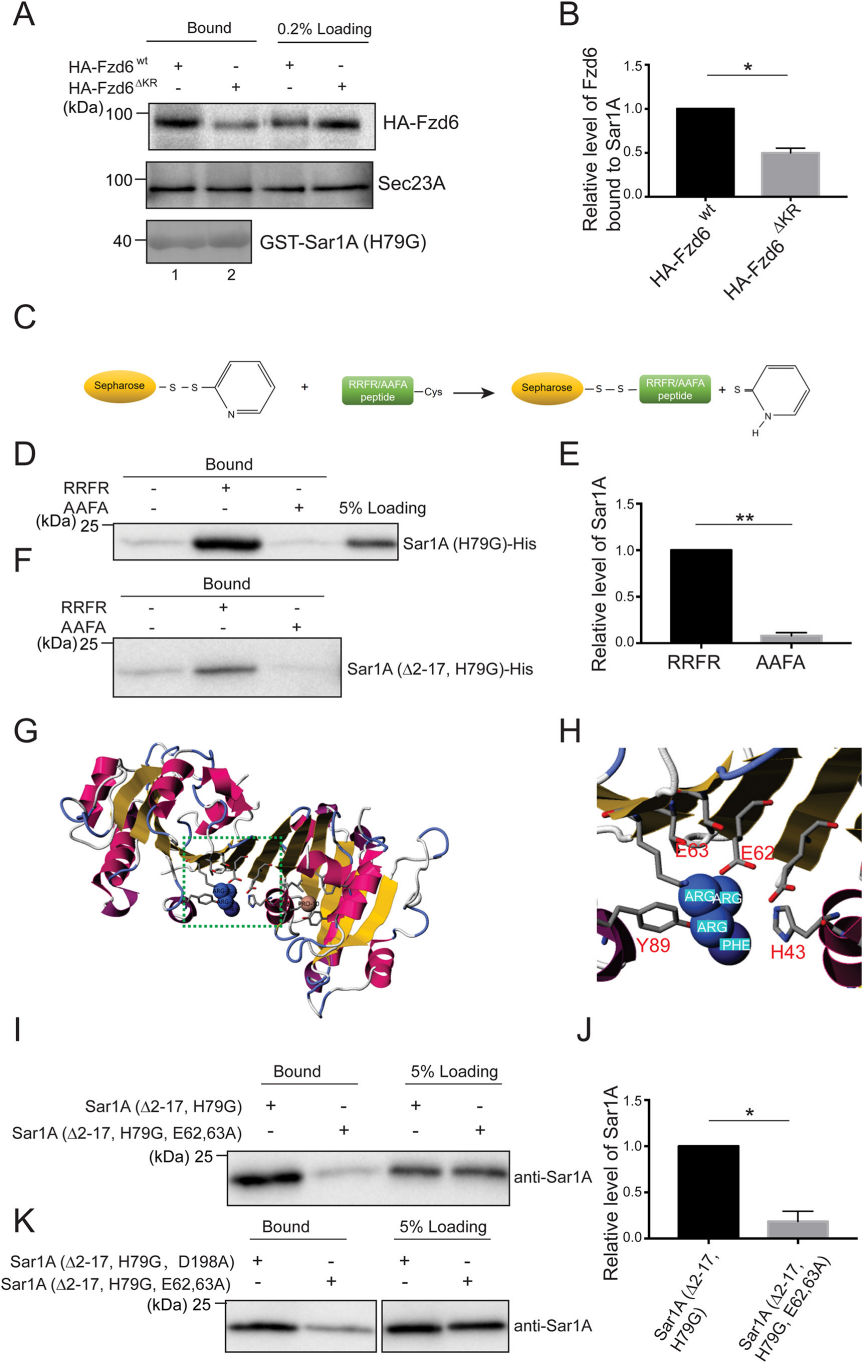
**The polybasic motif in Fzd6 directly interacts with the E62 and E63 residues on human Sar1A.** *A*, purified GST-tagged human Sar1A (H79G) was loaded with GTPγS, incubated with lysates from HEK293T cells transfected with HA-Fzd6^wt^ or HA-Fzd6^ΔKR^. After incubation, the bound proteins were analyzed by immunoblotting. *B*, relative levels of HA-Fzd6^WT^ or HA-Fzd6^ΔKR^ that bound to GST-Sar1A (H79G) were quantified (*n* = 3, mean ± S.D.). The quantification is normalized to the level of HA-Fzd6^WT^ that bound to Sar1A in each experimental group. *, *p* < 0.05. *C–F*, RRFR peptides or AAFA peptides were covalently linked to thiopyridone-Sepharose 6B (*C*), incubated with purified His-tagged human Sar1A^H79G^ or His-tagged human Sar1A^Δ2-17, H79G^. After incubation, the bound proteins were analyzed by immunoblotting (*D* and *F*). Levels of Sar1A^H79G^ bound to the RRFR peptide or AAFA peptide were quantified (*n* = 3, mean ± S.D.) (*E*). The quantification is normalized to the level of Sar1A that bound to RRFR peptides in each experimental group. **, *p* < 0.01. *G*, the structure of human Sar1A in complex with the RRFR peptide predicted by PepSite 2. *H*, a magnified view of the indicated area in *panel G*. *I–K*, RRFR peptides were covalently linked to thiopyridone-Sepharose 6B beads, incubated with Sar1A (Δ2-17, H79G)-His, Sar1A (Δ2-17, E62A, E63A, H79G), or Sar1A (Δ2-17, H79G, D198A). After incubation, the bound proteins were analyzed by immunoblotting (*I* and *K*). Levels of Sar1A (Δ2-17, H79G) or Sar1A (Δ2-17, E62A, E63A, H79G) that bound to RRFR peptides were quantified (*n* = 3, mean ± S.D.) (*J*). The quantification is normalized to the level of Sar1A (Δ2-17, H79G)-His that bound to RRFR peptides in each experimental group. *, *p* < 0.05.

To test whether Fzd6 interacts with COPII components, we performed GST pulldown experiments using recombinant purified GST-tagged Sar1A (H79G) (GST-Sar1A^H79G^) and lysates from HEK293T cells expressing HA-Fzd6^WT^ or HA-Fzd6^ΔKR^. GST-Sar1A^H79G^ interacted with HA-Fzd6^WT^ in cell lysates (Fig. 3*A*). Mutating the polybasic motifs significantly reduced the affinity between Sar1A and Fzd6 (Fig. 3*A* and *B*). The immobilized peptides corresponding to the first intracellular loop of Fzd6 (VRRFRYPERP, referred to as RRFR peptides) bound robustly to purified His-tagged Sar1A (H79G) (Sar1A^H79G^-His) (Fig. 3*C* and *D*). In contrast, alanine substitution of the RRFR peptides (VAAFAYPERP, referred to as AAFA peptides) blocked the binding (Fig. 3*D* and *E*). Purified Sar1A^H79G^-His depleted of its N-terminal amphipathic helix (Sar1A^Δ2-17, H79G^-His) also bound to the RRFR peptides but not AAFA peptides (Fig. 3*F*). These results suggest that the COPII subunit Sar1A directly interacts with the RRFR motif in the first intracellular loop of Fzd6.

Structural analysis indicates that purified His-tagged hamster and human Sar1A in complex with GDP form a dimer (17). We used PepSite 2 (18) to predict RRFR peptide binding sites on human Sar1A based on the crystal structure of human Sar1A (PDB ID 2GAO). The prediction indicates that the RRFR peptides bind at the interface of the Sar1A dimer (Fig. 3*G* and *H*). The E62 and E63 residues on Sar1A are predicted to interact with the Arg residues in RRFR peptides through electrostatic interactions (Fig. 3*H*). In addition, the side chains of H43 and Y89 on Sar1A can interact with the Phe residue in RRFR peptides through hydrophobic interactions (Fig. 3*H*). Mutating the E62 and E63 residues caused a significant reduction of the affinity between the RRFR peptides and Sar1A (Fig. 3*I* and *J*). These results indicate that the RRFR motif in Fzd6 directly interacts with the E62 and E63 residues on Sar1A.

The C terminus of many Golgi-resident glycosyltransferases contains a dibasic motif that is important for exporting these Golgi enzymes from the ER (19, 20). The D198 residue on Sar1 is identified to regulate its binding to the cytoplasmic tail of glycosyltransferases containing dibasic motifs (21). Interestingly, we found that Sar1A bearing the E62A and E63A mutations showed a much weaker affinity to the RRFR peptides than Sar1A bearing the D198A mutation (Fig. 3*K*), suggesting that RRFR peptides preferentially bind to the E62 and E63 residues on Sar1A.

### The direct interaction between the polybasic motif of Fzd6 and Sar1A is important for packaging of Fzd6 into COPII vesicles

**Figure 4.**
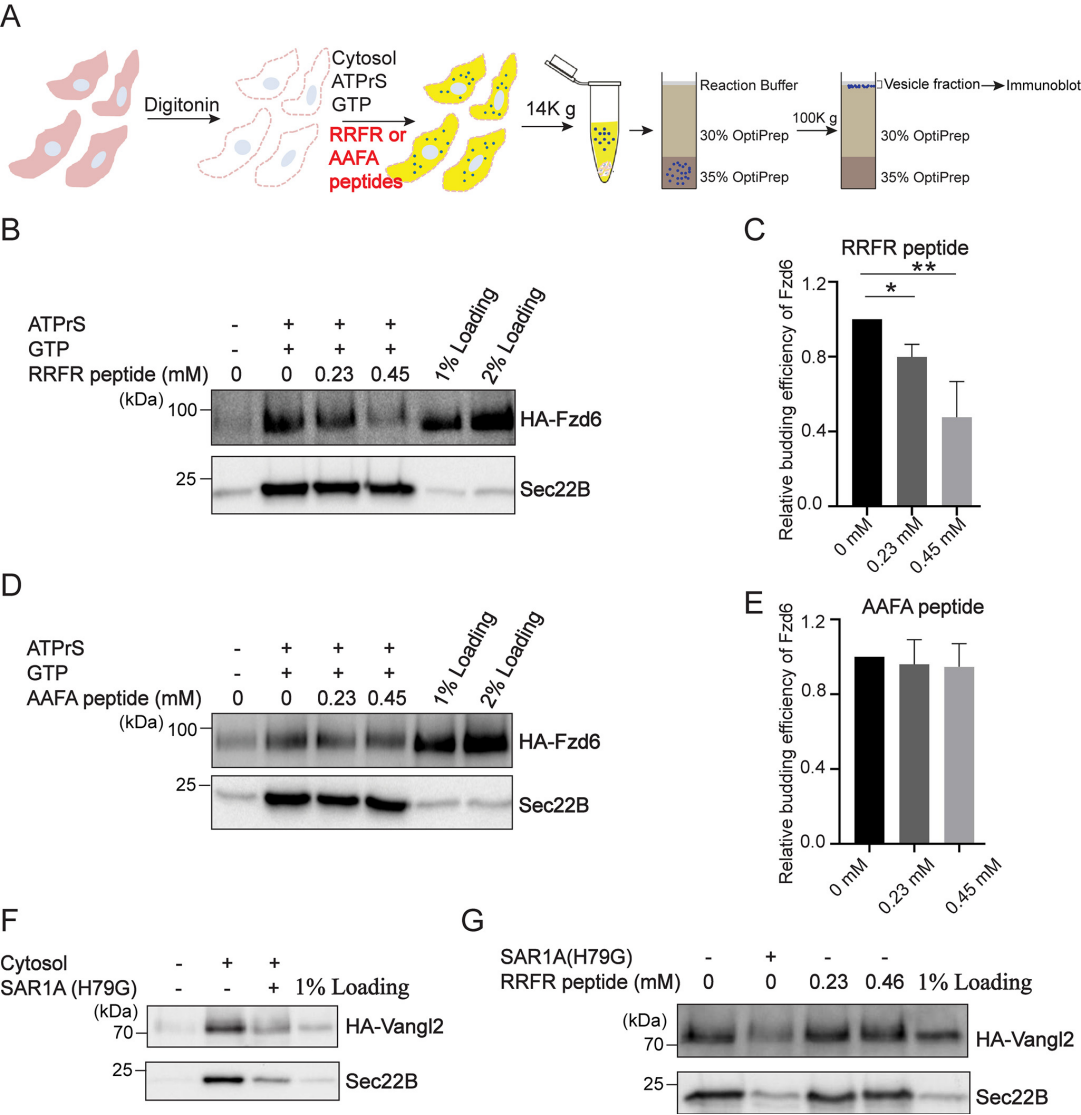
**The peptides corresponding to the first intracellular loop of Fzd6 inhibit the packaging of Fzd6 into COPII vesicles.** *A*, a diagram showing the vesicle formation assay performed in the presence of the synthesized peptides. *B–E*, the vesicle formation assay was performed using HEK293T cells expressing WT HA-Fzd6 in the presence of the RRFR peptides (*B*) or the AAFA peptides in which the polybasic motif is mutated (*D*). Vesicle fractions were then analyzed by immunoblotting. The budding efficiency of HA-Fzd6 in the groups with different concentrations of peptides were quantified (*C* and *E*) (*n* = 3, mean ± S.D.). The quantification is normalized to the level of Fzd6 in the vesicle fraction from the experimental group performed without peptide. *, *p* < 0.05; **, *p* < 0.01. *F–G*, the vesicle formation assay was performed using HEK293T cells expressing WT HA-Vangl2 in the presence of the reagents indicated.

We hypothesized that a peptide corresponding to the RRFR peptides can function as an inhibitor to block the interaction between Sar1A and Fzd6, thereby inhibiting the packaging of Fzd6 into COPII vesicles. To test this, we performed the vesicle formation assay in the presence of RRFR peptides or AAFA peptides (Fig. 4*A*). We found that RRFR peptides blocked vesicular release of Fzd6 but not Sec22B in a concentration-dependent manner (Fig. 4*B* and *C*). In contrast, AAFA peptides did not inhibit vesicular release of Fzd6 (Fig. 4*D* and *E*). COPII-mediated vesicular release of another PCP protein, Vangl2, has been reconstituted in COS7 cells (11). We reconstituted COPII-mediated vesicular release of Vangl2 in HEK293T cells (Fig. 4*F*). We found that vesicular release of Vangl2 was not inhibited by RRFR peptides (Fig. 4*G*), suggesting that sorting of Vangl2 into COPII vesicles was mediated by a mechanism that is different from Fzd6.

**Figure 5.**
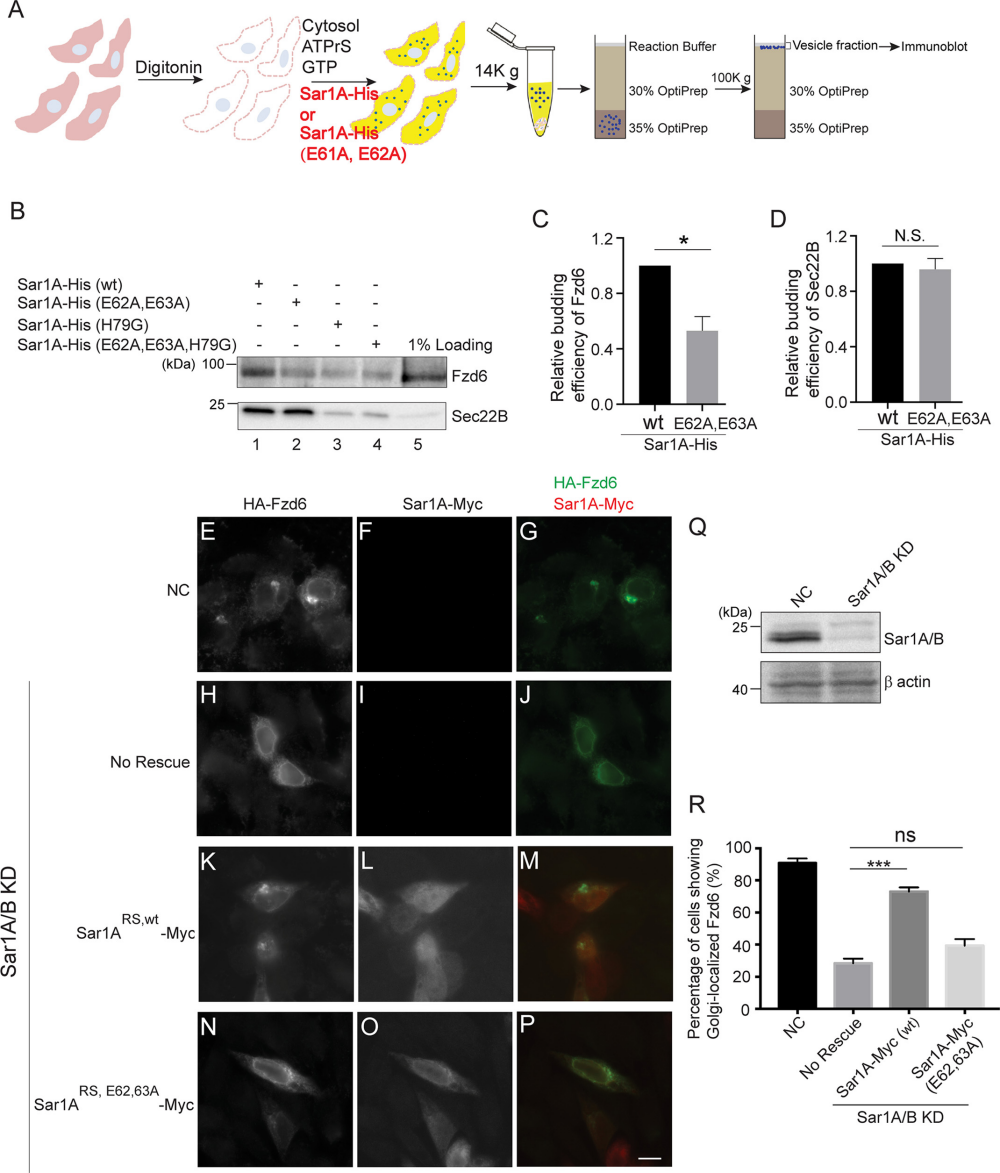
**The E61, E62 residues in human Sar1A are important for the packaging of Fzd6 into COPII vesicles to be delivered to the Golgi.** *A*, a diagram showing the vesicle formation assay performed in the presence of purified Sar1A-His or Sar1A^E61A, E62A^-His. *B*, the vesicle formation assay was performed using HEK293T cells expressing HA-Fzd6 using the indicated reagents. *C–D*, the budding efficiency of HA-Fzd6 and Sec22B from the vesicle formation assay performed in the presence of Sar1A-His or Sar1A ^E61A, E62A^-His was quantified (*n* = 3, mean ± S.D.). *, *p* < 0.05; N.S., not significant. The quantification is normalized to the level of Fzd6 or Sec22B from the assay performed in the presence of Sar1A-His. *E–P*, HeLa cells were mock transfected (*E–G*) or transfected with siRNA against Sar1A and Sar1B (*H–P*) and retransfected after 48 h with plasmids encoding HA-Fzd6 (*E–J*), retransfected with plasmids encoding the siRNA-resistant Sar1A^RS,wt^-Myc and HA-Fzd6 (*K–M*), or retransfected with plasmids encoding the siRNA-resistant Sar1A^RS,E62,63A^-Myc and HA-Fzd6 (*N–P*). On day 3 after knockdown, cells were incubated at 20°C for 2 h and analyzed by immunofluorescence. *Size bar*, 10 μm. *Q*, the levels of Sar1A/B in control cells or cells treated with siRNA against Sar1A and Sar1B were analyzed by immunoblotting. *R*, percentage of cells showing Golgi-localized Fzd6 was quantified (*n* = 3, mean ± S.D., over 100 cells were quantified in each experimental group). ***, *p* < 0.001.

As an additional approach to test whether the Fzd6-Sar1A interaction is important for ER export of Fzd6, we performed the vesicle formation assay in the presence of purified His-tagged Sar1A^E62A, E63A^ to test whether Sar1A^E62A, E63A^-His affects the efficiency of release of Fzd6 into transport vesicles (Fig. 5*A*). As observed previously, we detected Sar1A^H79G^-His, which greatly reduced the abundance of Fzd6 and Sec22B into transport vesicles (Fig. 5*B*, compare *lanes 1 and 3*). When the vesicle formation assay was performed in the presence of Sar1A^E62A, E63A^-His, we detected a significant reduction of the abundance of Fzd6 in the vesicle fraction (Fig. 5*B*, compare *lanes 1 and 2*, Fig. 5*C*). In contrast, the level of Sec22B was not affected by Sar1A^E62A, E63A^-His (Fig. 5*B*, compare *lanes 1 and 2*, Fig. 5*D*). These results indicate that the E62 and E63 residues on Sar1A are important for the packaging of Fzd6 into COPII vesicles.

We then tested whether the E62 and E63 residues on Sar1A are important for the delivery of Fzd6 from the ER to the Golgi *in vivo*. Fzd6 was located mainly at the ER in HeLa cells at steady state. When cells were incubated at 20 °C to block cargo export from the TGN, around 85% of cells showed Fzd6 located at the juxtanuclear Golgi area (Fig. 5*E*–*G* and *R*). Blocking ER export by knocking down the expression of Sar1A and Sar1B caused a significant reduction in the percentage of cells showing Golgi-localized Fzd6 after incubation at 20 °C (Fig. 5*H*–*J* and *Q–R*). Based on this observation, we hypothesized that incubation at 20 °C did not affect the efficiency of ER export but reduced the efficiency of TGN export, thereby causing an accumulation of Fzd6 in the Golgi. Expression of an siRNA-resistant construct of Sar1A (Sar1A^RS,wt^-Myc) rescued the percentage of Fzd6 in the Golgi under these conditions (Fig. 5*K*–*M* and *R*). In contrast, expression of an siRNA-resistant construct of Sar1A bearing E62A and E63A mutations (Sar1A^RS, E62, E63A^-Myc) did not rescue Fzd6 (Fig. 5*N*–*P* and *R*), suggesting that the E62 and E63 residues on Sar1A are important for the delivery of Fzd6 from the ER to the Golgi. These analyses indicate that a direct interaction between the polybasic motifs of Fzd6 and Sar1A is important for the packaging of Fzd6 into COPII vesicles to be delivered to the Golgi.

### *Celsr1 associates with Fzd6 in the secretory transport pathway*, *and this association regulates their surface delivery process*

**Figure 6.**
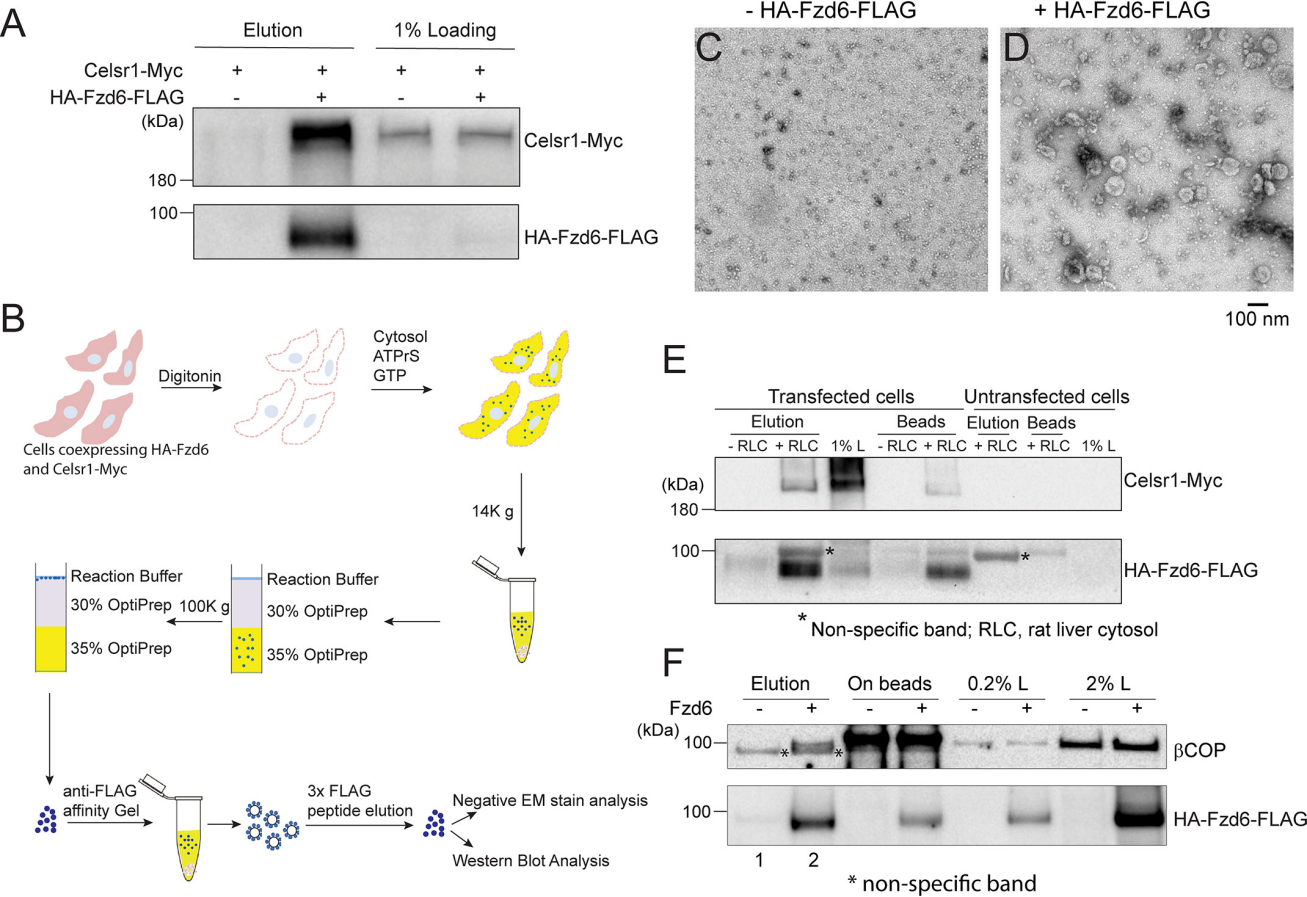
**Celsr1 associates with Fzd6 in the secretory transport pathway.** *A*, lysates from HEK293T cells transfected with HA-Fzd6-FLAG and Celsr1-Myc were incubated with anti-FLAG affinity gel. The bound proteins were eluted by FLAG peptides, and the eluted fractions were analyzed by immunoblotting. *B*, a diagram showing the procedures to isolate Fzd6-enriched vesicles. *C–D*, negative-stain EM analysis of the immune-isolated vesicles from untransfected cells (*C*) or from cells transfected with plasmids encoding HA-Fzd6-FLAG (*D*). *E*, Fzd6-enriched vesicles were isolated using HEK293T cells cotransfected with HA-Fzd6-FLAG and Celsr1-Myc. The eluted vesicles were analyzed by immunoblotting. *F*, Fzd6-enriched vesicles were isolated from untransfected HEK293T cells or cells transfected with plasmids encoding HA-Fzd6-FLAG and then analyzed by immunoblotting.

Celsr1, also known as Flamingo in *Drosophila*, is one of the core PCP proteins. Celsr1 is a seven-pass transmembrane protein and locates to both the distal and the proximal sides of cell boundaries. In *Drosophila*, evidence suggests that Flamingo proteins form homodimers at cell boundaries and recruit Frizzled and Vang to the opposing cell junctions, contributing to the establishment of PCP asymmetry (3). To test whether Celsr1 and Fzd6 interact with each other, we performed coimmunoprecipitation experiments using HEK293T cells cotransfected with plasmids encoding Celsr1-Myc and an HA-tagged Fzd6 construct containing a FLAG tag in the C-terminal cytosolic domain (HA-Fzd6-FLAG) (9). We found that Celsr1-Myc coimmunoprecipitated with HA-Fzd6-FLAG (Fig. 6*A*), suggesting that Celsr1 interacted with Fzd6 in mammalian cells. Next, we tested whether Celsr1 and Fzd6 are associated with each other in the same transport vesicles. We performed the vesicle formation assay at a large scale using HEK293T cells transfected with plasmids encoding HA-Fzd6-FLAG. The vesicle fractions were then incubated with agarose beads conjugated with mouse anti-FLAG antibodies. Vesicles that bound to beads were eluted using FLAG peptides and analyzed by negative-stain EM and immunoblotting (Fig. 6*B*). Untransfected HEK293T cells were used as a negative control. We detected small membrane-bound structures in the immune-isolated fraction from the HA-Fzd6-FLAG group (Fig. 6*D*) but not in the control group (Fig. 6*C*). We previously demonstrated that immune-isolated Fzd6 vesicles produced from cells coexpressing Vangl2 and Fzd6 do not contain Vangl2, suggesting these two PCP proteins are present in separate vesicles (9). Here, we performed the assay using cells coexpressing HA-Fzd6-FLAG and Celsr1-Myc, and we found that immune-isolated Fzd6 vesicles contain Celsr1-Myc (Fig. 6*E*). These results indicate that Fzd6 and Celsr1 are in the same transport vesicles.

**Figure 7.**
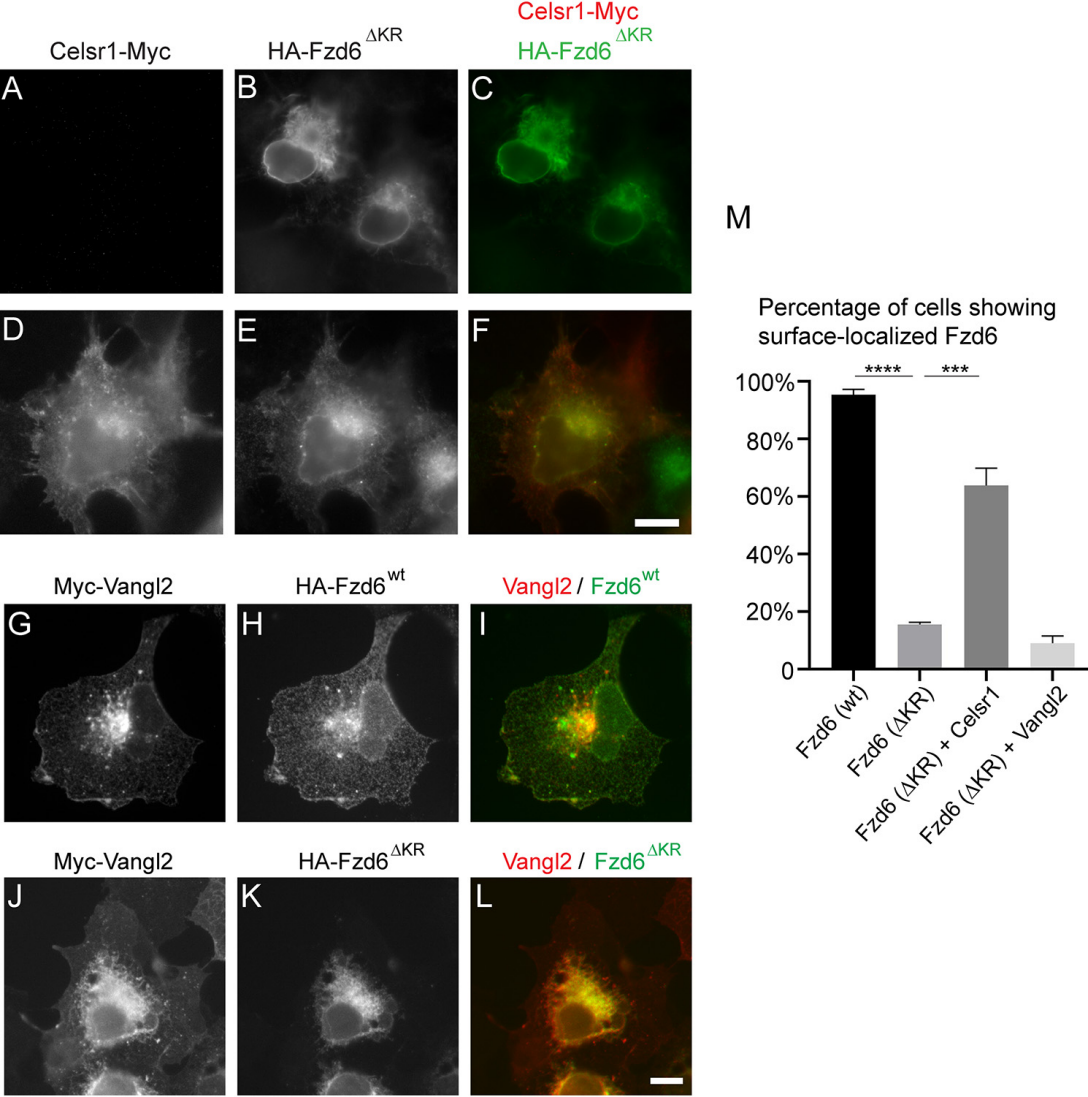
**Association of Celsr1 with Fzd6 partially rescues ER export defects of the COPII-binding-deficient mutant form of Fzd6.** *A–L*, COS7 cells were transfected with HA-Fzd6^ΔKR^ (*A–C*), cotransfected with HA-Fzd6^ΔKR^ and Celsr1-Myc (*D–F*), cotransfected with Myc-Vangl2 and HA-Fzd6^wt^ (*G–I*), or cotransfected with Myc-Vangl2 and HA-Fzd6^ΔKR^ (*J–L*). Day 1 after transfection, localizations of the indicated proteins were analyzed by immunofluorescence. *Size bar*, 10 μm. *M*, quantification analyses of the percentage of cells showing surface-localized Fzd6 in cells expressing the indicated constructs (*n* = 3, mean ± S.D., over 80 cells were quantified in each experimental group). ***, *p* < 0.001; ****, *p* < 0.0001.

We analyzed whether the association of Fzd6 and Celsr1 is important for surface delivery of Fzd6 in COS7 cells. To test this, we coexpressed Celsr1-Myc and HA-Fzd6^ΔKR^ in COS7 cells and analyzed their localizations. HA-Fzd6^ΔKR^, when expressed alone, accumulated at the ER (Fig. 7*A*–*C*). Interestingly, this construct, when coexpressed with Celsr1-Myc, was partially localized at the plasma membrane and colocalized with Celsr1 in the majority of the coexpression cells (Fig. 7*D*–*F*). Quantification analysis indicates that the percentage of cells showing surface-localized HA-Fzd6^ΔKR^ in cells coexpressing Celsr1-Myc was significantly higher than that detected in cells not expressing Celsr1-Myc (Fig. 7*M*). Coexpressing another PCP protein, Myc-Vangl2, did not increase the percentage of cells showing surface-localized HA-Fzd6^ΔKR^ (Fig. 7*G*–*L*, *M*). These results suggest that the association of Celsr1 with Fzd6 partially rescued ER export defects of the COPII-binding-deficient mutant form of Fzd6.

**Figure 8.**
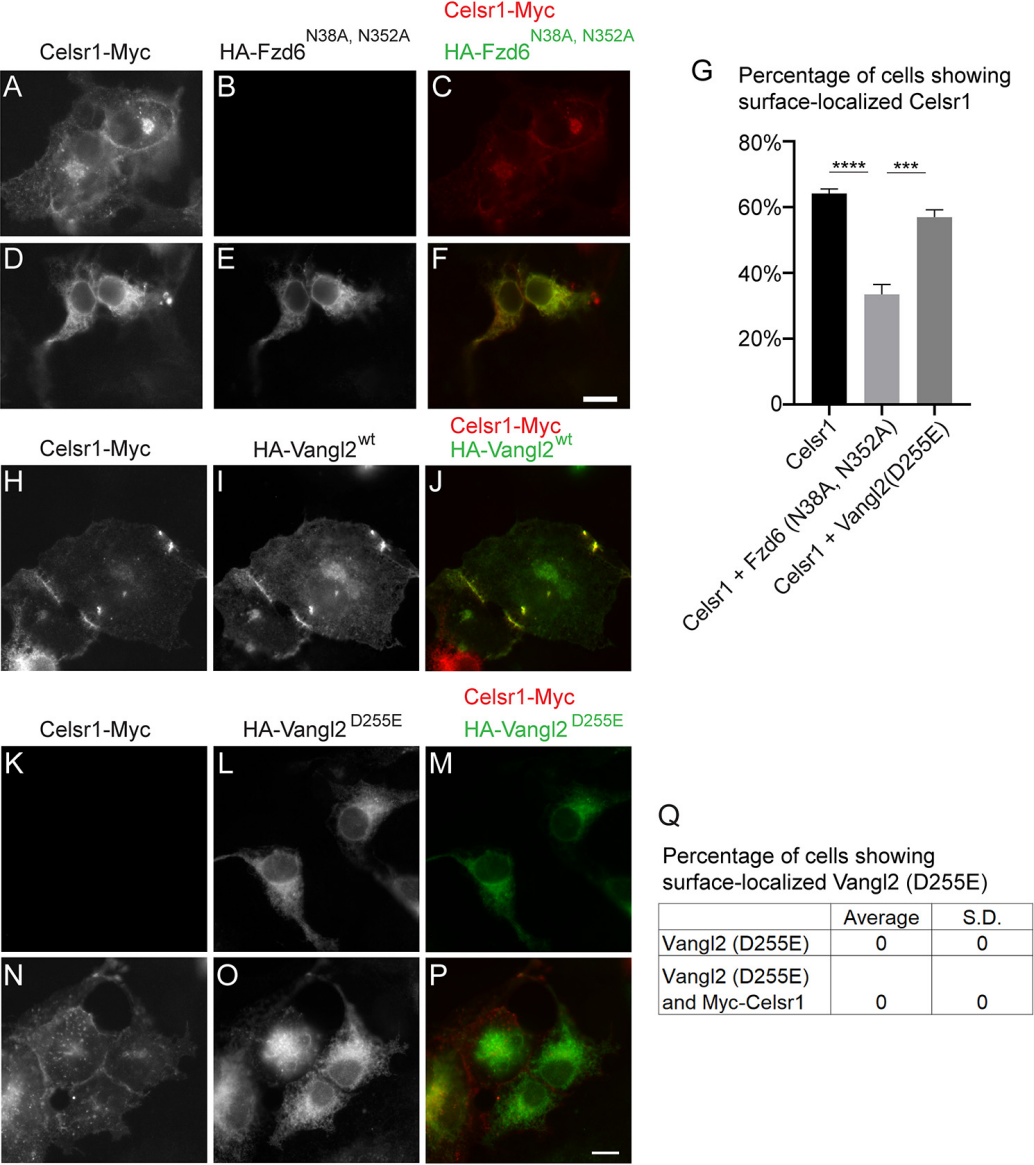
**Retention of the N-glycosylation-defective mutant Fzd6^N38A, N352A^ at the ER causes ER retention of Celsr1.** *A–F* and *H–P*, COS7 cells were transfected with Celsr1-Myc (*A–C*), transfected with HA-Vangl2^D255E^ (*K–M*), cotransfected with HA-Fzd6^N38A, N352A^ and Celsr1-Myc (*D–F*), cotransfected with HA-Vang2^wt^ and Celsr1-Myc (*H–J*), or cotransfected with HA-Vangl2^D255E^ and Celsr1-Myc (*N–P*). Day 1 after transfection, localizations of the indicated proteins were analyzed by immunofluorescence. *Size bar*, 10 μm. *G* and *Q*, quantification analyses of the percentage of cells showing surface-localized Celsr1 and Vangl2 ^D255E^ in cells expressing the indicated constructs (*n* = 3, mean ± S.D., over 80 cells were quantified in each experimental group). ***, *p* < 0.001; ****, *p* < 0.0001.

Frizzled family proteins undergo N-glycosylation modifications. In *Xenopus* embryos, the immature glycosylated form of Frizzled interacts with Shisa, causing ER retention of Frizzled (12). We found that the luminal domain of Fzd6 contains two predicted N-glycosylation sites, N38 and N352 (Fig. S1*A*). HA-tagged WT Fzd6 (HA-Fzd6^wt^) was sensitive to peptide:*N*-glycosidase F digestion (Fig. S1*B*, compare *lanes 1 and 2*). In contrast, the mutant version of Fzd6, in which these two predicted N-glycosylation sites were replaced with alanine (HA-Fzd6^N38A, N352A^), was resistant to peptide:*N*-glycosidase F digestion (Fig. S1*B*, compare *lanes 1 and 2*), suggesting that these two residues are important for N-glycosylation modifications on Fzd6. HA-Fzd6^wt^ was partially located at the plasma membrane (Fig. S1*C–E*), whereas HA-Fzd6^N38A, N352A^ showed an ER-localized pattern (Fig. S1*F–H*). In addition, the efficiency of release of Fzd6^N38A, N352A^ into vesicles was lower than the efficiency of vesicular release of Fzd6^WT^ (Fig. S1*I*, compare lanes 2 and 6). This result indicates that Fzd6^N38A, N352A^ is defective for export out of the ER, possibly by interacting with an ER-resident protein. Interestingly, when Fzd6^N38A, N352A^ was coexpressed with Celsr1-Myc, Celsr1-Myc accumulated at the ER with no detectable surface pattern in the majority of the coexpression cells (Fig. 8*A*–*F*). Quantification analysis indicates that the percentage of cells showing surface-localized Celsr1-Myc in cells coexpressing Fzd6^N38A, N352A^ was significantly lower than that detected in cells not expressing Fzd6^N38A, N352A^ (Fig. 8*G*), and the percentage was also significantly lower than that detected in cells coexpressing Celsr1-Myc and an HA-Vangl2 loop tail mutant, HA-Vangl2^D255E^ (Fig. 8*G*). These analyses suggest that retention of Fzd6 at the ER causes ER retention of Celsr1.

Next, we analyzed whether accumulations of another PCP protein, Vangl2, at the ER causes ER retention of Celsr1 in COS7 cells. In mouse keratinocyte monolayers, WT Vangl2 was colocalized with Celsr1 at cell junctions when two neighboring cells both were coexpressing these two constructs (22). A similar localization pattern was detected in COS7 cells coexpressing HA-tagged WT Vangl2 and Celsr1-Myc (Fig. 8*H*–*J*). HA-Vangl2^D255E^ was located at the ER with no detectable surface pattern (11) (Fig. 8*L*). The surface-localized HA-Vangl2^D255E^ was not detectable in the coexpression cells (Fig. 8*N*–*P*, quantification in *Q*), suggesting that Celsr1 did not rescue the defects of surface delivery of Vangl2 ^D255E^. In addition, the surface-localized Celsr1-Myc was clearly detectable in cells coexpressing HA-Vangl2^D255E^ and Celsr1-Myc (Fig. 8*N*), suggesting that accumulations of Vangl2^D255E^ at the ER do not cause ER retention of Ceslr1.

**Figure 9.**
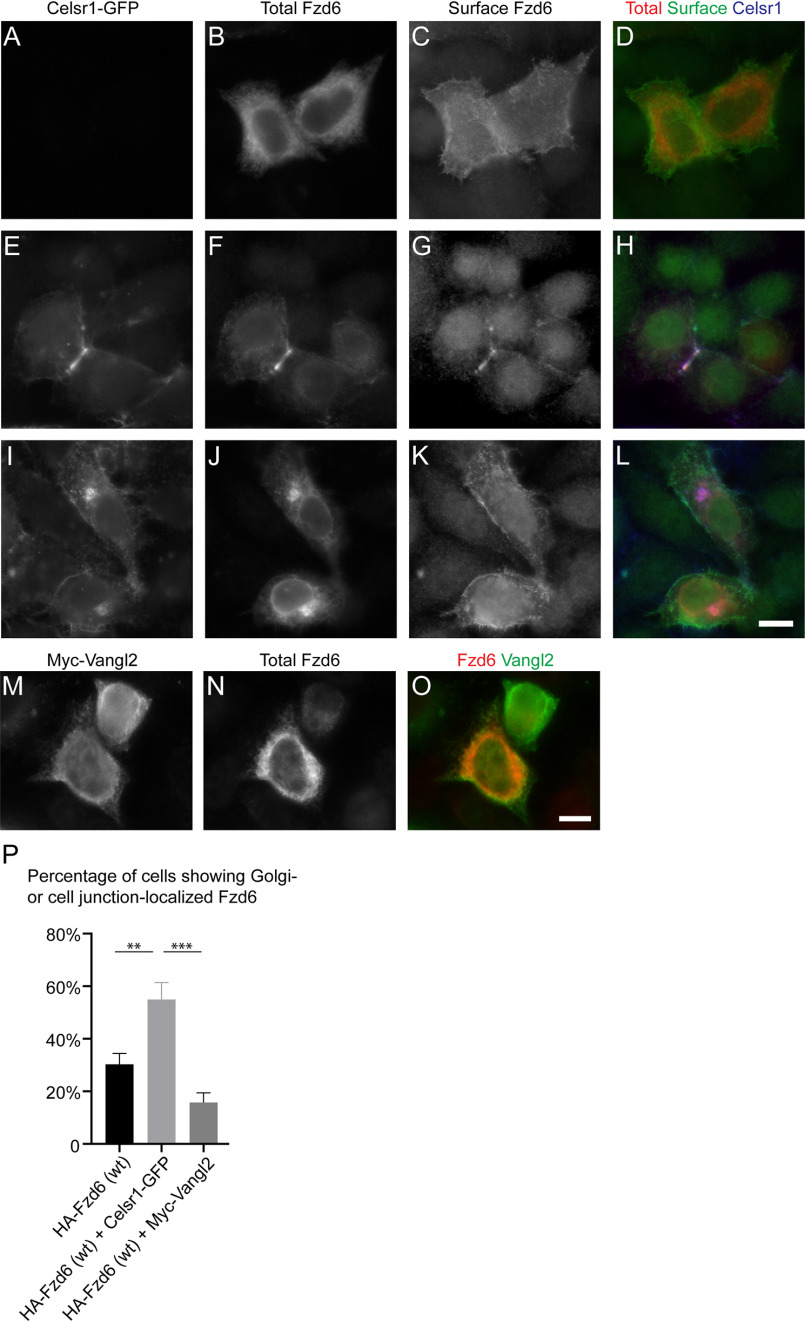
**Celsr1 promotes the anterograde trafficking of Fzd6 in HeLa cells.** *A–O*, HeLa cells were cotransfected with Celsr1-GFP and HA-Fzd6 (*A–L*) or cotransfected with Myc-Vangl2 and HA-Fzd6 (*M–O*). Day 1 after transfection, localizations of the indicated proteins were analyzed by immunofluorescence. The surface-localized HA-Fzd6 and total HA-Fzd6 were stained by mouse and rabbit anti-HA antibodies, respectively. *Size bar*, 10 μm. *P*, percentage of cells showing Golgi- or cell junction-localized Fzd6 was quantified (*n* = 3, mean ± S.D., over 60 cells were quantified in each experimental group). **, *p* < 0.01; ***, *p* < 0.001.

We then analyzed whether the association of Fzd6 and Celsr1 is important for surface delivery of Fzd6 in HeLa cells in addition to COS7 cells. In HeLa cells, Fzd6 showed an obvious ER-localized pattern at steady state in the majority of cells (Fig. 9*B*). The surface-localized Fzd6 was detected by surface labeling of the HA tag present at the extracellular domain of Fzd6 (Fig. 9*C*–*D*). Over 80% of cells showed strong accumulations of Fzd6 in the Golgi area after incubation at 20 °C (Fig. 5*R*), suggesting that Fzd6 can be efficiently exported out of the ER. We found that the COPI coat subunit, βCOP, was detected in the immune-isolated Fzd6 vesicles (Fig. 6*F*, *lane 2*), suggesting that the COPI coat regulates Golgi-to-ER retrieval of Fzd6. Interestingly, Fzd6 strongly accumulated at the cell junctions colocalized with Celsr1 in many cells coexpressing Fzd6 and Celsr1 (Fig. 9*E*–*H*). A similar observation also was observed in *Drosophila* pupal wing and in mouse keratinocyte monolayers (5, 22). In some cells that coexpressed Celsr1 and Fzd6, Fzd6 accumulated at the juxtanuclear Golgi area (Fig. 9*I*–*L*). Quantification analysis indicates that the percentage of cells showing Golgi- or cell junction-localized Fzd6 in the cells coexpressing Celsr1 was significantly higher than that detected in the cells that are not expressing Celsr1 (Fig. 9*P*). Coexpressing Myc-Vangl2 did not increase the percentage of cells showing Golgi- or cell junction-localized Fzd6 (Fig. 9*M*–*P*). These results indicate that Celsr1 promotes anterograde trafficking of Fzd6.

### The polybasic motif of Frizzled is important for its surface localization in Drosophila wing

**Figure 10.**
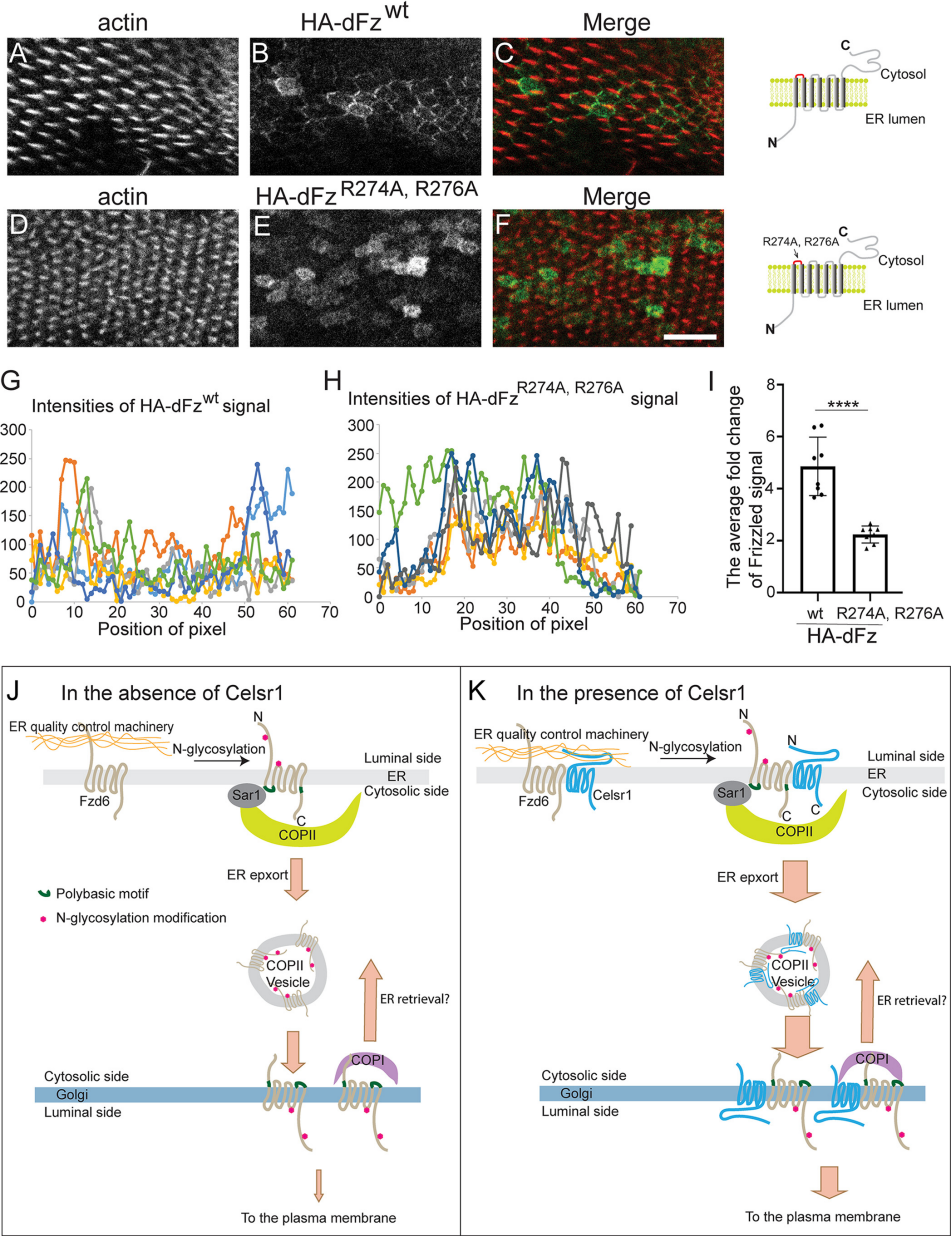
**The polybasic motif of dFz is important for its surface localization in *Drosophila* wing.** *A–F*, pupa wings expressing dFz^wt^ (*B*) or dFz^R274A, R276A^ were stained to visualize actin and HA-tagged dFz. *Size bar*, 10 μm. *G–H*, examples showing the intensity of dFz signal along each line that crosses a cell. *I*, the fold change of the highest intensity of dFz signal along each line that crosses a cell over the intensity of dFz signal in the middle point of the line was quantified (mean ± S.D., based on 8 random fields; each field contains over 7 cells in each experimental group). ****, *p* < 0.0001. *J–K*, our proposed model describing the molecular mechanisms regulating delivery of Fzd6 from the ER to the Golgi.

Frizzled in *Drosophila* contains an RFR motif in its first intracellular loop but no polybasic motifs in its C-terminal cytosolic tail. We generated transgenic fly strains with UAS-HA-tagged *Drosophila* Frizzled (HA-dFz^wt^) and UAS-HA-tagged *Drosophila* Frizzled, in which the first and second arginine residues of the RFR motif in the first intracellular loop were mutated to alanine (HA-dFz^R274A, R276A^). Using the engrailed-Gal4 (en-Gal4, en>) driver to specifically induce the expression of HA-dFz^wt^ or HA-dFz^R274A, R276A^ in the posterior territory of the wing disc, we found that HA-dFz^wt^ was located on cell boundaries in *Drosophila* wing disc cells (Fig. 10*A*–*C*). In contrast, HA-dFz^R274A, R276A^ was largely localized intracellularly (Fig. 10*D*–*F*). Figure 10*G*–*H* demonstrated the profiles of intensities of dFz signal along lines that cross six randomly selected cells expressing HA-dFz^wt^ or HA-dFz^R274A, R276A^. HA-dFz^wt^ on the cell boundaries showed the highest intensities and were clearly detected in each profile. In contrast, HA-dFz^R274A, R276A^ on the cell boundaries cannot be clearly detected in the majority of the profiles. We quantified the fold change of the highest intensity of dFz signal along each line that crosses a cell over the intensity of dFz signal in the middle point of the line. The average fold change detected from cells expressing HA-dFz^R274A, R276A^ was significantly reduced compared to that detected from cells expressing HA-dFz^wt^ (Fig. 10*I*). These results indicate that the polybasic motif (RFR) of dFz is important for its surface localization in *Drosophila* wing disc cells.

## Discussion

The ER is the first station in the secretory transport pathway. Regulating the ER export process can affect the surface delivery of newly synthesized signal receptors, thereby influencing the intracellular signaling events. In this study, we demonstrated that ER export of Fzd6 is regulated by several steps. An Fzd6 construct bearing mutations in its N-glycosylation sites accumulated in the ER, possibly by interaction with an ER-resident protein in the ER quality control machinery (Fig. 10*J*). After correct folding and N-glycosylation modification, Fzd6 will escape from the ER quality control machinery. Subsequently, the polybasic motif located on the first intracellular loop on Fzd6 directly interacts with the E62 and E63 residues on Sar1A, and this interaction regulates the packaging of Fzd6 into COPII vesicles (Fig. 10*J*). The polybasic motif on dFz is important for surface localization of dFz in *Drosophila* wing, suggesting that this motif is important for surface delivery of Frizzled in animals. At the Golgi, COPI coat may function to mediate Golgi-to-ER retrieval of Fzd6 (Fig. 10*J*). We propose that the efficiency of COPII-mediated ER export of WT Fzd6 is higher than the efficiency of COPI-mediated ER retrieval, which causes WT Fzd6 to be productively delivered to the downstream compartments along the secretory transport pathway. Our analysis indicates that the efficiency packaging of Fzd6^ΔKR^ into COPII vesicles is significantly lower than that of WT HA-Fzd6 (Fig. 2*B* and *C*). Thus, we hypothesize that the efficiency of ER export of Fzd6^ΔKR^ is lower than the efficiency of COPI-mediated ER retrieval, which causes Fzd6^ΔKR^ to be located at the ER at the steady state.

The polybasic motif has been shown to bind multiple binding partners in addition to Sar1. A triple arginine motif in the third intracellular loop of the α_2B_-adrenergic receptor (α_2B_-AR) regulates ER export of α_2B_-AR and interacts with Sec24C/D isoforms (23). It will be interesting to analyze whether Fzd6 is also recognized by the Sec24C/D isoforms. We recently found that a conserved polybasic motif on the Fzd6 C-terminal cytosolic domain is recognized by the clathrin adaptor epsinR, and this interaction is important for the TGN export process (9). The polybasic motif of the reptilian reovirus p14 protein mediates the interaction between activated Rab11 and p14 and regulates TGN export of p14 (24, 25). We propose that the polybasic motif interacts with its binding partner through electrostatic interactions, which provide a major force for binding but not, by itself, determining the binding specificity. The region surrounding the polybasic motif may also play important roles for determining the binding specificity or affinity. In this study, we found that the phenylalanine residue in RRFR peptides can form hydrophobic interactions with the adjacent histidine and tyrosine residues on Sar1A (Fig. 3*H*), which may contribute to the affinity between RRFR peptides and Sar1A.

Transmembrane core PCP proteins interact with each other intercellularly on the opposing cell boundaries. The interactions between these PCP proteins play important roles in stabilizing the asymmetric junctional complexes and in the propagation of PCP (3, 5, 6, 26, 27). In addition, transmembrane core PCP proteins also interact with specific cytosolic core PCP components intracellularly. The interacellular and intracellular interactions among core PCP proteins promote the two asymmetrically located PCP complexes to be localized at mutually exclusive regions at opposite sides of each cell (6). As such, up- or downregulation of any core PCP proteins causes defects in the localization of other core PCP components (6, 28, 29). Our results suggest that Celsr1 and Fzd6 associate with each other in the secretory transport pathway (Fig. 10*K*). When associated with the immaturely glycosylated form of Fzd6, Clesr1 is retained in the ER (Fig. 10*K*). After correct glycosylation and folding, Fzd6 in association with Celsr1 is released from the ER quality control machinery and then interacts with the COPII machinery to be packaged into COPII vesicles. At this step, Celsr1 promotes the anterograde transport of Fzd6, possibly by providing additional binding sites for COPII (Fig. 10*K*). These analyses provide an additional function of the association between Celsr1 and Fzd6. Our study also provides a mechanism whereby Frizzled proteins that are associated with Celsr1 can be effectively delivered from the ER to the Golgi and the plasma membrane, suggesting a quality control mechanism to ensure the stoichiometry of Fzd6 and Celsr1 on cell boundaries.

## Materials and methods

### *Constructs*, *reagents*, *cell culture*, *immunofluorescence*, *and transfection*

The plasmids encoding HA-tagged mouse Vangl2, HA-tagged mouse Fzd6, and HA-Fzd6-FLAG were generated as described previously (9). The plasmids encoding HA-Fzd6^R225A, R226A^, HA-Fzd6^△508-513, R225A, R226A^, and HA-Fzd6^N38A, N352A^ were generated by QuikChange II site-directed mutagenesis using plasmids encoding HA-Fzd6-FLAG as a template. The plasmids encoding GST-tagged human Sar1A and His-tagged human Sar1A were generated as described previously (9). The plasmids encoding GST-Sar1A (H79G) and Sar1A (H79G)-His were generated by QuikChange II site-directed mutagenesis. The plasmids encoding Sar1A (Δ2-17, H79G)-His were generated by cloning Sar1A (Δ2-17, H79G) amplified from Sar1A (H79G)-His into pET-28a. The plasmids encoding Sar1A (H79G, E62A, E63A)-His, Sar1A (E62A, E63A)-His, and Sar1A (Δ2-17, H79G, E62A, E63A)-His were generated by QuikChange II site-directed mutagenesis. The plasmids encoding pTIGER-dFz-HA and pTIGER-dFz (R274A, R276A)-HA were ordered from BGI. The plasmids encoding Myc-tagged mouse Celsr1 and Myc-tagged Vangl2 were generously provided by the Devenport laboratory (Princeton, NJ).

siRNAs against Sar1A and Sar1B were purchased from Ribobio (Guangzhou, China). The target sequence against Sar1A is GGAATGACCTTTACAACTT. The target sequence against Sar1B is CTGGTAAACTGGTATTTCT. The commercial antibodies were rabbit anti-HA (Cell Signaling, catalogue number 3724), sheep anti-TGN46 (AbD Serotec, catalogue number AHP500G), mouse anti-Myc (Cell Signaling, catalogue number 2276), mouse anti-PDI (Enzo, catalogue number ADI-SPA-891-F), mouse anti-actin (Proteintech, catalogue number 60008-1). Rabbit anti-Sar1A/B, rabbit anti-Sec22B, and rabbit anti-Sec23A were kindly provided by Prof. Randy Schekman (University of California, Berkeley, CA, USA). Peptides used in the *in vitro* vesicle formation assays and pulldown assays were purchased from GenScript (Nanjing, China).

HeLa cells and HEK293T cell lines were kindly provided by the University of California-Berkeley Cell Culture Facility and were confirmed by short tandem repeat profiling. COS7 cells were obtained from the ATCC (catalog number ATCC CRL-1651, RRID:CVCL_0224). All cell lines tested negative for *Mycoplasma* contamination. HeLa, HEK293T, and COS7 cells were maintained in GIBCO Dulbecco's modified Eagle's medium containing 10% fetal bovine serum (FBS), 10 milliunits/ml penicillin, and 0.1 mg/ml streptomycin. Transfection of siRNA or DNA constructs into HeLa cells, HEK293T cells, or COS7 cells and immunofluorescence were performed as described previously (7, 9). A temperature shift experiment was performed as described previously (9). Images were acquired with a Zeiss Axio Observer Z1 microscope system (Carl Zeiss, Jena, Germany) equipped with an ORCA Flash 4.0 camera (Hamamatsu, Hamamatsu, Japan).

### *Immunoprecipitation*, *protein purification*, *and binding assay*

Immunoprecipitation of FLAG-tagged Fzd6 was performed by incubating 10 μl of compact anti-FLAG M2-agarose affinity beads with 200 μl of 0.5 mg/ml cell lysates from HEK293T cells transfected with HA-Fzd6-FLAG in HKT buffer (100 mm KCl, 20 mm Hepes, pH 7.2, 0.5% Triton X-100) with mixing at 4 °C overnight. After incubation, the beads were washed 4 times with 1 ml of HK buffer (100 mm KCl, 20 mm Hepes, pH 7.2), and the bound material was eluted with 30 μl of 0.6 mg/ml FLAG peptides in HK buffer (100 mm KCl, 20 mm Hepes, pH 7.2) at 4 °C overnight.

Purification of GST-tagged Sar1A and His-tagged Sar1A were performed as described previously (7, 9). Binding assays were carried out with 10 μl of compact GSH beads bearing around 5 μg of GST-tagged Sar1A (H79G). The beads were incubated with 200 μl of 0.5 mg/ml of cell lysates from HEK293T cells transfected with HA-Fzd6 or HA-Fzd6^△508-513, R225A, R226A^ in HKT buffer with mixing at 4 °C overnight. After incubation, the beads were washed four times with 500 μl of HK buffer, and the bound material was analyzed by immunoblotting.

Synthetic RRFR peptides (VRRFRYPERPC) or AAFA peptides (VAAFAYPERPC) were purchased from GenScript and coupled to thiopyridone-Sepharose 6B beads (Sigma-Aldrich) via the added C-terminal cysteine residue. The coupling reaction was performed based on previous reports (19, 20). For binding experiments, 2 μg purified His-tagged Sar1A was preincubated at 4 °C for 30 min with 500 μm GTPγS in a total volume of 15 μl HK buffer. After incubation, 15 μl buffer containing around 5 μl beads containing 5 nmol of peptides was added to the reaction mixture for 1 h at 4 °C. The beads were washed four times with 500 μl of HK buffer and analyzed by immunoblotting.

### The vesicle formation assay

*In vitro* vesicular release assays were performed using HEK293T cells. The approach was a modified version of the vesicle budding reaction designed previously to detect COPII vesicle formation at the ER (11, 13, 14). Day 1 after transfection with plasmids encoding HA-tagged Fzd6 constructs or HA-Vangl2, cells grown in one 10-cm dish at around 90% confluence were permeabilized in 3 ml of ice-cold KOAc buffer (110 mm potassium acetate, 20 mm Hepes, pH 7.2, 2 mm magnesium acetate) containing 40 μg/ml digitonin on ice for 5 min, and the semi-intact cells were then sedimented by centrifugation at 300 × *g* for 3 min at 4 °C. The cell pellets were washed twice with 1 ml of KOAc buffer and resuspended in 100 μl of KOAc buffer. The budding assay was performed by incubating semi-intact cells (around 0.02 OD/reaction) with 2 mg/ml of rat liver cytosol in a 100-μl reaction mixture containing 200 μm GTP and an ATP regeneration system (40 mm creatine phosphate, 0.2 mg/ml of creatine phosphokinase, and 1 mm ATP) in the presence or absence of 0.5 μg of Sar1A, Sar1A (H79G), or Sar1A (E62A, E63A) mutant protein. After incubation at 32 °C for 1 h, the reaction mixture was centrifuged at 14,000 × *g* to remove cell debris and large membranes. The medium-speed supernatant was then centrifuged at 100,000 × *g* to sediment small vesicles. The pellet fraction was then analyzed by immunoblotting. For density gradient flotation assays, the pellet fraction was resuspended in 100 μl of 35% OptiPrep and overlaid with 700 μl of 30% OptiPrep and 30 μl of KOAc buffer. The samples were centrifuged at 55,000 rpm in a TLS55 rotor in a Beckman ultracentrifuge for 2 h at 4 °C. After centrifugation, fractions were collected from the top to the bottom of the tube, and the top fraction was analyzed by SDS-PAGE and immunoblotting.

To immunoisolate vesicles enriched with Fzd6, the volume of the reaction mixture was scaled up to 1.3 ml. The medium-speed supernatant was then incubated with 30 μl of compact anti-FLAG M2-agarose affinity beads at 4 °C overnight. After incubation, the beads were washed 4 times with 1 ml of KOAc buffer and eluted with 50 μl of KOAc buffer containing 0.6 mg/ml of FLAG peptides. The eluted fraction was then analyzed by immunoblotting or negative-stain EM. Negative electron microscopy analysis was performed according to a previous report (9).

### Drosophila stocks and Drosophila histology

The animal study was approved by the Animal Ethics Committee at HKUST. Transgenic flies expressing UAS-HA-dFz^wt^ or UAS-HA-dFz^R274A, R276A^ were generated by WellGenetics Inc. (Taipei, Taiwan). Engrailed-Gal4 was obtained from the Bloomington *Drosophila* Stock Center (BL25752). Flies were raised on standard medium and maintained at 25 °C, unless otherwise indicated. The GAL4/UAS system was used in this study for gene expression. For analysis of pupal wings, prepupae (white pupae) were collected and staged at 25 °C for 30–32 h after pupae formation. Wings were dissected, fixed, and stained by following a standard protocol (30). HA antibody was purchased from Cell Signaling (1:250, #3724). The secondary antibody conjugated with Alexa Fluor dye (1:500) and Alexa-Phalloidin (1:1,000) were purchased from ThermoFisher. Pupal wing images were acquired at room temperature using a Leica SP8.

## Data availability

All data are contained within the article and accompanying supporting information.

## Supplementary Material

Supporting Information
