# Supplementary material for: Molecular mechanisms that regulate export of the planar cell-polarity protein Frizzled-6 out of the endoplasmic reticulum
Source: J Biol Chem. 2020 May 6;295(27):8972–87. doi: 10.1074/jbc.RA120.012835 (PMC7335806; doi:10.1074/jbc.RA120.012835)
Supplement: Supporting Information [file supp_RA120.012835_58500_2_supp_525686_q9x9mp.pdf]

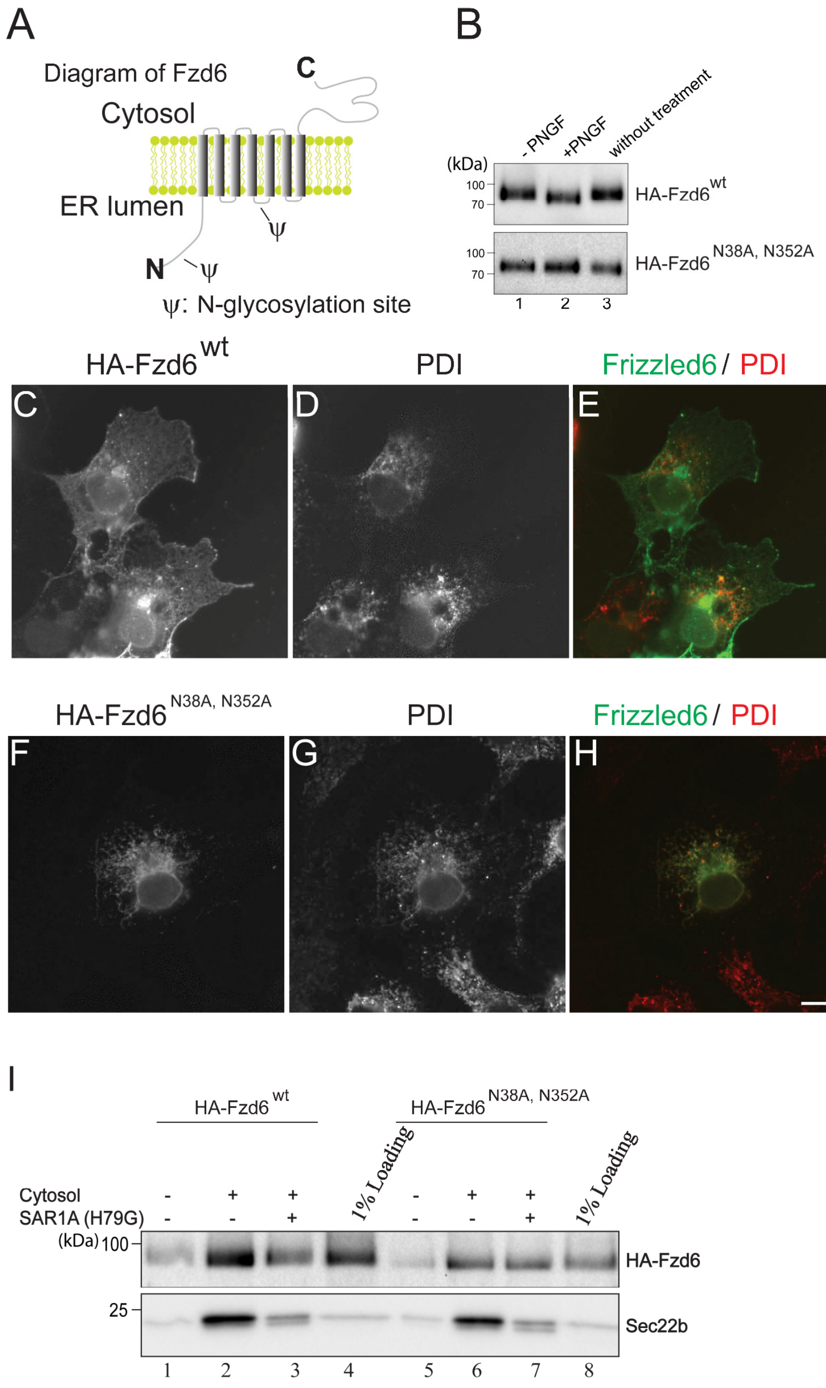

Figure S1. ER export of Fzd6 depends on the N-glycosylation motif.

A. A diagram of wide type Fzd6 with two predicted N-glycosylation sites. **B.** HA-Fzd6 or HA-Fzd6$^{N38A, N352A}$ immune-precipitated from COS7 cell lysates were untreated (lane 3) or incubated with a reaction mixture in the presence or absence of PNG-F (lanes 1 and 2) and then analyzed by immunoblot. **C-H.** COS7 cells were transfected with wild type HA-Fzd6 (C-E), HA-Fzd6 bearing mutations in its N-glycosylation sites (HA-Fzd6 $^{N38A, N352A}$, F-H). At day 1 after transfection, the cells were analyzed by immunofluorescence. Size bar, 10 µm. **I.** The vesicle formation assay was performed using HEK293T cells expressing wild type HA-Fzd6 (HA-Fzd6^{WT}) or HA-Fzd6$^{N38A, N352A}$. Vesicle fractions were analyzed by immunoblotting with the indicated antibodies. PNGF, peptide:N-glycosidase F.
